# Axonal Injury Induces ATF3 in Specific Populations of Sacral Preganglionic Neurons in Male Rats

**DOI:** 10.3389/fnins.2018.00766

**Published:** 2018-10-24

**Authors:** Agnes W. Wong, Peregrine B. Osborne, Janet R. Keast

**Affiliations:** Department of Anatomy and Neuroscience, University of Melbourne, Melbourne, VIC, Australia

**Keywords:** parasympathetic, intermediolateral nucleus, preganglionic neurons, axotomy, axon regeneration, spinal nerve injury

## Abstract

Compared to other neurons of the central nervous system, autonomic preganglionic neurons are unusual because most of their axon lies in the periphery. These axons are vulnerable to injury during surgical procedures, yet in comparison to peripheral neurons and somatic motor neurons, the impact of injury on preganglionic neurons is poorly understood. Here, we have investigated the impact of axotomy on sacral preganglionic neurons, a functionally diverse group of neurons required for micturition, defecation, and sexual function. We have previously observed that after axotomy, the injury-related transcription factor activating transcription factor-3 (ATF3) is upregulated in only half of these neurons (Peddie and Keast, 2011: PMID: 21283532). In the current study, we have investigated if this response is constrained to particular subclasses of preganglionic neurons that have specific functions or signaling properties. Seven days after unilateral pelvic nerve transection, we quantified sacral preganglionic neurons expressing ATF3, many but not all of which co-expressed c-Jun. This response was independent of soma size. Subclasses of sacral preganglionic neurons expressed combinations of somatostatin, calbindin, and neurokinin-1 receptor, each of which showed a similar response to injury. We also found that in contrast to thoracolumbar preganglionic neurons, the heat shock protein-25 (Hsp25) was not detected in naive sacral preganglionic neurons but was upregulated in many of these neurons after axotomy; the majority of these Hsp25 neurons expressed ATF3. Together, these studies reveal the molecular complexity of sacral preganglionic neurons and their responses to injury. The simultaneous upregulation of Hsp25 and ATF3 may indicate a distinct mechanism of regenerative capacity after injury.

## Introduction

Autonomic preganglionic neurons are the first relay in peripheral visceral motor pathways. They are central nervous system (CNS) neurons with a soma located in either spinal cord or brainstem, but like somatomotor neurons are unusual in that most of the axon projects outside of the CNS. These long axons project through peripheral nerves to autonomic postganglionic neurons, making them vulnerable to physical damage. This can occur where they exit from the CNS (spinal nerve or spinal cord damage) or closer to the organs, where they are vulnerable to surgical procedures, such as removal of tumors. This axonal damage can severely disrupt autonomic motor function, but in comparison with somatic motor pathways, the impact of injury on preganglionic neurons is poorly understood.

Parasympathetic regulation of urogenital organs and the lower bowel is provided by sacral preganglionic neurons found in the intermediolateral nucleus (IML) below the lumbar spinal cord enlargement. By convention, they are identified as sacral preganglionic neurons, but in rodents are found in L6 and S1 spinal cord segments (Hancock and Peveto, 1979). Functional activation of these pathways is required for the erectile response of the penis or clitoris, micturition, defecation, and secretion from various glandular cells of the reproductive tract. After exiting the spinal cord, sacral preganglionic axons travel together in the pelvic nerves (rodents) or pelvic splanchnic nerves (humans), which is consistent with the profound impact on many pelvic organ functions caused by damage to these nerves, as seen in conus medullaris and cauda equina syndromes (Orendacova et al., 2001; Hoang and Havton, 2006; Hoang et al., 2006).

Pelvic nerve injury in rodents has been used as an experimental model to study how sacral preganglionic neurons respond to mechanical damage of their peripheral axons. For example, we have reported (Peddie and Keast, 2011) that pelvic nerve transection does not cause neuronal death but can induce somatic changes in a proportion of the preganglionic neurons in the sacral IML ipsilateral to the injury, such as reduced expression of choline acetyltransferase (ChAT). The classical “injury response” markers, c-Jun and ATF3, are also upregulated, but only in and around half of the injured preganglionic neurons (Peddie and Keast, 2011). This suggests that a major population of these neurons do not use the immediate-early gene transcription factors c-Jun and ATF3 as master regulators of the injury response and regenerative growth in axons (Jenkins et al., 1993; Herdegen et al., 1997; Tsujino et al., 2000; Averill et al., 2004; Raivich et al., 2004; Lindwall and Kanje, 2005; Hyatt Sachs et al., 2007). Furthermore, this raises the possibility that certain molecular or functional classes of sacral preganglionic neurons have reduced susceptibility to injury and could in turn have increased capacity for regeneration.

In this study, we have used unilateral pelvic nerve transection in male rats to determine if different effects of axotomy can be detected in immunohistochemically defined classes of sacral preganglionic neurons identified by combinatorial expression of neurokinin 1 receptor (NK1R), somatostatin (SOM), calbindin (CAL), or heat shock protein-25 (Hsp25). NK1R is the preferred receptor for substance P. A major class of visceral afferents that project to pelvic organs and express this sensory neuropeptide (Keast and de Groat, 1992; Arms and Vizzard, 2011) have central terminals in both the IML as well as the dorsal horn of the sacral spinal cord (Sasek et al., 1984; Vizzard, 2001; Zhang et al., 2008). NK1R is also expressed by many thoracolumbar preganglionic neurons (Cammack and Logan, 1996; Grkovic and Anderson, 1996; Parsons et al., 1996; Burman et al., 2001; Ishigooka et al., 2001). Somatostatin (SOM) is also widely expressed in autonomic preganglionic neurons (Dalsgaard et al., 1981; Krukoff et al., 1985, 1986; Samano et al., 2006). In the sacral spinal cord it is expressed by a subpopulation of preganglionic neurons that regulate the bladder and bowel, but is rarely found in preganglionic neurons that regulate erectile tissue (Keast and de Groat, 1989; Keast, 1991, 1994). The calcium binding protein, calbindin, is expressed in subpopulations of thoracolumbar preganglionic neurons (Antal et al., 1990; Zhang et al., 1990; Ren and Ruda, 1994; Grkovic and Anderson, 1997; Merkulyeva et al., 2015) and in some neurons this is suggested to correlate with survival after axotomy (Krebs et al., 1997; Dassesse et al., 1998; Obal et al., 2006). Hsp25 is expressed by many thoracolumbar preganglionic neurons (Plumier et al., 1997) and regulated by injury, when it promotes survival (Costigan et al., 1998; Lewis et al., 1999). To our knowledge, Hsp25 and CAL have not previously been examined specifically in rodent sacral preganglionic neurons.

ATF3 was used in this study as a marker to identify neurons undergoing a post-injury regenerative response. It was used in preference to the immediate-early gene marker c-Jun, which can also be induced by non-injury stimuli such as deafferentation (Nangle and Keast, 2009). For each molecular subclass of sacral preganglionic neurons, we have quantified the proportion expressing ATF3, potentially indicating the neurons undergoing a regenerative response. To optimize the quantitation of sacral preganglionic neurons we have used an intraperitoneal injection of FluoroGold to identify the total population (Anderson and Edwards, 1994), rather than relying on a neural marker such as ChAT that is downregulated by injury (Peddie and Keast, 2011; Coulibaly et al., 2013).

## Materials and Methods

### Animals and Reagents

All procedures were approved by the Animal Ethics Committee of the University of Melbourne and complied with the Australian Code for the Care and Use of Animals for Scientific Purposes (National Health and Medical Research Council of Australia). Twenty-one adult male Sprague–Dawley rats (6–9 weeks of age) were used for this study, supplied by the Biomedical Sciences Animal Facility at the University of Melbourne. Twelve of these underwent unilateral pelvic nerve transection surgery and in nine animals tissue was removed without any other intervention (naive group). Rats were housed in groups of three with environmental enrichment under a 12-h light–dark cycle and free access to food and water. Chemicals were obtained from Sigma-Aldrich (St. Louis, MO, United States) unless otherwise indicated.

### Surgery

Each animal first received an intraperitoneal injection (300 μl of a 0.5% solution in sterile saline) of the retrograde tracer, FluoroGold (FG; Fluorochrome, Englewood, CO, United States). Administration by this route labels all neurons that have axons extending out of the CNS to the abdominal and pelvic organs, including all sacral preganglionic neurons (Anderson and Edwards, 1994; Peddie and Keast, 2011). Seven days later, unilateral pelvic nerve transection was performed under isoflurane anesthesia (3% for induction, and 1.8–2% for maintenance, in O_2_). Transection was performed <1 mm from the major pelvic ganglion and a 1–2 mm segment of pelvic nerve was removed to prevent regeneration. The pelvic nerve was visualized on the contralateral side, but no lesion was performed. Analgesic (buprenorphine; 0.05 mg/kg in 0.05 ml saline, subcutaneous) was administered once at the start of the surgery and again ∼10 h after surgery. Tissues were removed from these rats seven days after surgery, as per below; this time was chosen on the basis of our earlier study that showed reduced ATF3 and c-Jun expression after this time (Peddie and Keast, 2011). No health issues arose in the postoperative period.

### Tissue Preparation

Naive rats or rats that had undergone pelvic nerve transection were anesthetized by i.p. injection of ketamine (100 mg/kg) and xylazine (10 mg/kg), then intracardially perfused with approximately 100 ml of normal saline solution (containing 1% sodium nitrite and 5,000 IU/ml heparin), followed by approximately 500 ml of 4% paraformaldehyde in 0.1 M phosphate buffer, pH 7.4. The lumbosacral cord was then dissected and stored overnight in the same fixative, washed in 0.1 M phosphate-buffered saline (PBS), pH 7.2, and the region including the full extent of both L6 and S1 removed for further study. The spinal cord specimen was then marked to differentiate ipsilateral and contralateral sides and incubated overnight in 0.1 M PBS containing 30% sucrose before embedding in an inert mounting medium (OCT; Tissue-Tek, Sakura, Torrance, CA, United States) and cryosectioning at a thickness of 40 μm. Sections from a 1 in 4 series were then processed for fluorescence immunohistochemistry. All analyses were performed in sections cut in the horizontal (i.e., coronal) orientation to enable the columnar structure of the IML to be visualized along its rostrocaudal axis. This provides much more efficient sampling of IML neurons than the more commonly studied transverse sections.

### Immunohistochemistry

Free-floating sections of the spinal cord were first incubated in 0.1 M PBS (pH 7.2) containing 10% non-immune horse serum (NHS; Sigma-Aldrich). To characterize expression patterns in preganglionic neurons, sections were incubated for 48–72 h at room temperature with the following combinations of primary antisera (antibody details below): ChAT (1:500) with either SOM (1:2,000) or CAL (1:500), or NK1R (1:2,000) with CAL (1:500). Each antibody combination was prepared in PBS containing 0.1% sodium azide, 2% NHS, and 0.5% Triton X-100. After washes in PBS, sections were then incubated for 4 h at room temperature with appropriate combinations of biotin-labeled donkey anti-goat (1:300), AF488-labeled donkey anti-rabbit (1:1,000), and AF594-labeled donkey anti-mouse (1:1,000). After further washes in PBS, sections labeled with the biotin-tagged secondary antibody were incubated for 1 h at room temperature with streptavidin-AMCA (1:300). Sections were then washed in PBS, mounted onto glass slides, and cover-slipped with phosphate-buffered glycerol (pH 8.6).

To co-label SOM and NK1R, a different staining protocol was required because both antisera were raised in the same host species. First, immunolabeling for SOM was performed as described above for the primary incubation step, then followed by incubation with AF594-labeled donkey anti-rabbit (1:1,000). This was followed by NK1R immunofluorescence using a higher dilution of antibody and signal enhancement with tyramide amplification (TSA plus biotin kit; Perkin Elmer, cat # NEL749A001KT). After SOM immunohistochemistry, sections were incubated for 15 min in 2% bovine serum albumin in TBS buffer (supplied in the TSA kit), then with NK1R antibody (1:25,000) for 48 h. After a 15-min wash in BSA/PBS, sections were incubated in donkey anti-rabbit biotinylated antibody (4 h), followed by another BSA/PBS wash (15 min). They were then incubated with TSA reagent (TSA concentrate diluted 1:50 in TSA buffer provided with the kit; 1 h), washed again in BSA/PBS (15 min), and then anti-streptavidin-AF488 (1 h). After final washes in PBS, the sections were mounted as described above.

To examine the subpopulations of preganglionic neurons expressing the regeneration marker ATF3 after pelvic nerve transection, free-floating sections were incubated for 48–72 h in antisera against ATF3 (1:500) with SOM (1:2,000), CAL (1:500), or NK1R (1:2,000). After washes in PBS, the sections were subsequently incubated for 4 h at room temperature with donkey anti-rabbit AF488 (1:1,000) and donkey anti-mouse AF594 (1:1,000). Sections were then washed, mounted, and cover-slipped as above. Additional sections from these animals were incubated for 48 h in antisera containing ATF3 (1:500) with c-jun (1:20,000), or ATF3 with Hsp25 (1:500). After washes in PBS, the sections were incubated for 4 h at room temperature with donkey anti-rabbit AF488 (1:1,000) and donkey anti-mouse AF594 (1:1,000). Sections were then washed, mounted, and cover-slipped.

### Antibodies

Primary antibodies were obtained from the following sources, noting that information on specificity has been provided by the manufacturers:

1. ATF3 (Abcam, Cambridge, MA, United States; ab58668; batch GR95594-23): affinity purified mouse monoclonal antibody generated against recombinant C-terminal ATF3 peptide of human origin. Western blot analysis detects a band of approximately 35 kDa.
2. ATF3 [Santa Cruz Biotechnology, Inc., Santa Cruz, CA, United States; (C19) sc-188; batch J2209]: affinity purified polyclonal antibody generated in rabbit against an ATF3 C-terminal peptide of human origin. Western blot analysis of whole cell lysate detects a band at 27 kDa.
3. CAL (Sigma, St. Louis, MO, United States; C9848; batch 052M4833): monoclonal mouse antibody is derived from the BD-955 hybridoma produced by the fusion of mouse myeloma cells and splenocytes from BALB/c mice immunized with a purified bovine kidney calbindin-D-28K. Western blot analysis of MDBK cell extract detects a band at 28 kDa, consistent with the predicted weight.
4. ChAT (Millipore, Temecula, CA, United States; AB144P; batch 2695212): immunoaffinity purified polyclonal antibody generated in goat against the human placental enzyme. Western blot analysis of NIH/3T3 lysates detects a band at 70 kDa, consistent with the predicted weight of ChAT protein.
5. Hsp25 (Enzo Life Sciences, Farmingdale, NY, United States; ADI-SPA-801; batch 8071339): Affinity purified polyclonal antibody generated in rabbit from recombinant mouse Hsp25. Western blot detects a band of a predicted weight at 25 kDa.
6. c-Jun (Santa Cruz Biotechnology, Inc., Santa Cruz, CA, United States; sc-1694): polyclonal antibody generated in rabbit against amino acids 1–79 of human c-Jun. Western blot analysis detects a band at 43 kDa.
7. NK1R (Sigma, St. Louis, MO, United States; S8305; batch 067K4885): polyclonal antibody is generated in rabbit using as immunogen a synthetic peptide KTMTESSSFYSNMLA, corresponding to the C-terminus of NK1R of rat origin. Whole antiserum is purified to provide an IgG fraction of antiserum. Western blot analysis of rat brain lysate detects a band at predicted weight, 46 kDa.
8. SOM (Immunostar, Hudson, WI, United States; 20067; batch 8834021): polyclonal antibody generated in rabbit against synthetic somatostatin coupled to keyhole limpet hemocyanin with carbodiimide linker.

Secondary antisera were raised in donkey and obtained from the following suppliers:

1. Anti-goat biotin-SP (Jackson Immunoresearch, West Grove, PA, United States; 711-065-147; batch 116297)
2. Anti-rabbit biotin-SP (Jackson Immunoresearch; 711-065-152; batch 88458)
3. Anti-rabbit AF488 (Jackson Immunoresearch; 711-545-152; batch 126601)
4. Anti-rabbit AF594 (Jackson Immunoresearch; 711-585-152; batch 115265)
5. Anti-mouse AF594 (Molecular Probes; A21203; batch 1163390).

Other fluorescent tags used were: streptavidin-AF488 (Molecular Probes; S-11223; batch 84A2-2) and streptavidin-AMCA (Jackson Immunoresearch; 016-150-084; batch 111698).

### Quantitation of Neuronal Classes

Preganglionic neurons in the IML were identified by their location, expression of ChAT, and labeling by FG. To avoid double-counting neurons, only neurons sectioned through the nucleus were counted. Every 4th section was analyzed for each antibody combination. The prevalence of each type of preganglionic neuron was calculated as a proportion of the total number of sacral IML neurons, as indicated by ChAT in naive animals or FG in tissues from pelvic nerve transection studies. In sections where more than one neuronal marker was investigated, double-labeled neurons were expressed as a percentage of all neurons of that chemical class.

### Quantitation of Soma Profile Area

To compare the size of ATF3-positive and -negative preganglionic somata after injury, 8-bit monochrome images of the IML labeled for both ATF3 and ChAT were acquired using a Zeiss Axioimager M1 and MRm camera. Using Fiji software, the area of each nucleated soma profile was determined in a minimum of 20 neurons on each side of the IML per animal. In total, 135 IML neurons were measured ipsilateral to pelvic nerve transection and 105 contralateral in 4 rats.

### Statistical Analyses

To detect if pelvic nerve transection affected the proportions of neurons expressing each type of neural marker or neural soma size ipsilateral to injury, we performed paired, two-tailed Student’s *t*-tests. Statistical tests were performed using Prism 7 (Graphpad Software, La Jolla, CA, United States). Data are expressed as mean ± SEM, where *n* = number of rats.

### Figure Production

Monochrome images were digitally colorized and where necessary adjustments made in contrast and brightness to best represent the immunostaining as seen under the microscope (Adobe InDesign and Photoshop CC; Adobe Systems, San Jose, CA, United States).

## Results

### ATF3 and c-Jun Show Partial Coexpression in Injured Sacral Preganglionic Neurons

Our previous study focused primarily on characterizing c-Jun expression after axotomy of preganglionic neurons (Peddie and Keast, 2011). Although we also observed that this injury caused upregulation of ATF3, further characterization of these neurons was not performed. We prioritized this characterization here because ATF3 expression is so closely aligned to regenerative events after injury (see above).

**FIGURE 1:**
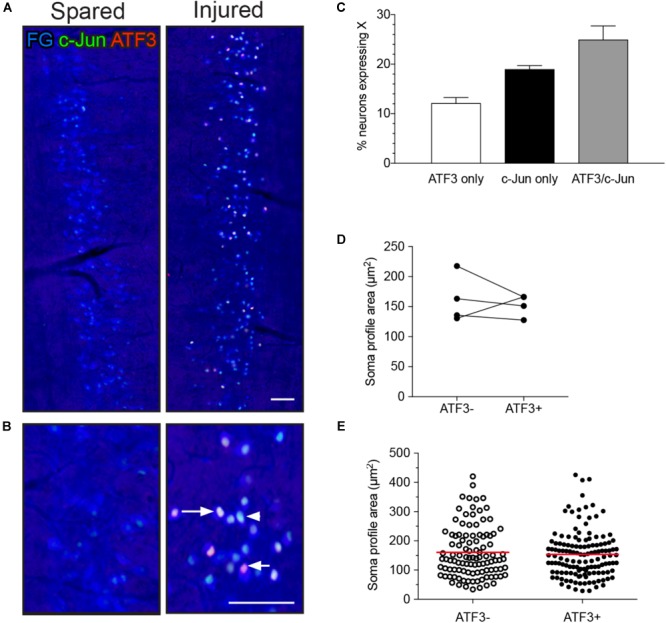
Expression of ATF3 and c-Jun in L6-S1 preganglionic neurons one week after unilateral transection of the pelvic nerve. Representative horizontal sections of spinal cord were immunolabeled for c-Jun (green) and ATF3 (red). Preganglionic neurons were identified by their uptake of retrograde tracer [FluoroGold (FG), here colorized blue]. Because all of the preganglionic neurons are quite intensely labeled by FluoroGold (shown as blue), the c-Jun nuclei appear turquoise rather than green and the ATF3 nuclei appear pink rather than red. Images are oriented with rostral at the top and lateral on the left (spared) or right (injured) of the relevant panel. **(A)** Contralateral to the injury (“spared”), no preganglionic neurons were immunoreactive for ATF3 and rare nuclei were immunoreactive for c-Jun; in contrast, ipsilateral to injury (“injured”) many preganglionic neurons were immunoreactive for ATF3, c-Jun, or both transcription factors (ATF3/c-Jun). An example of each is shown by an arrowhead (c-Jun), short arrow (ATF3), and long arrow (ATF3/c-Jun). **(B)** Higher magnification of spared (left) and injured (right) sides of the L6-S1 IML. **(C)** Quantitation of FG-positive preganglionic neurons ipsilateral to injury, showing the proportion that are immunoreactive for either ATF3 or c-Jun alone, or for both transcription factors (ATF3/c-Jun); together these comprise 56.1 ± 2.8% of sacral preganglionic neurons identified by FG (*n* = 6). **(D)** No difference was identified between the soma profile areas of neurons expressing ATF3 (153 ± 9 μm^2^) and ATF3-negative neurons (162 ± 20 μm^2^). Data shown as **(D)** mean soma profile area from each side of each animal and **(E)** individual neurons pooled from all animals. *n* = 4 rats, minimum of 20 neurons measured on each side in each rat, two-tailed, paired *t*-test: *P* = 0.65. Calibration bars represent 100 μm.

In L6-S1 spinal cord segments, ATF3 was absent and c-Jun was rarely expressed in the IML contralateral to pelvic nerve transection (Figures 1A,B; left panels) or on either side in the naive group (data not shown), confirming previous reports (Peddie and Keast, 2011; McCarthy et al., 2016). However, in the ipsilateral IML (Figures 1A,B; right panels), one or both transcription factors were expressed in the nuclei of a majority of the FG-labeled neurons (56.1 ± 2.8%, *n* = 6 rats; Figure 1C). All three classes of neurons (ATF3 with c-Jun, ATF3 only, and c-Jun only) appeared to be randomly distributed along the rostrocaudal axis of the IML.

Preganglionic neurons in L6-S1 IML are quite variable in size (Barber et al., 1984; Peddie and Keast, 2011) and transiently undergo a small decrease in volume after axotomy (Peddie and Keast, 2011). To determine if ATF3 is a marker for this reduction in volume, we measured the area of nucleated soma profiles of FG neurons in the IML ipsilateral to injury. This analysis failed to detect any significant difference in the mean soma size of ATF3-positive and -negative neurons (Figures 1D,E).

### Multiple Populations of Sacral Preganglionic Neurons Can Be Distinguished by SOM, CAL, and NK1R Expression Patterns

Spinal cords from the naive group were used to determine the immunohistochemical expression of SOM, CAL, and NK1R in sacral preganglionic neurons, which were identified by ChAT-immunoreactivity and their location in horizontal sections of L6-S1 spinal cord. As described in detail below, subgroups of ChAT neurons expressed one or more of SOM, CAL, or NK1R, but each neuron class appeared to be randomly distributed in the IML as no spatial pattern (e.g., rostrocaudal gradients) was detected.

**FIGURE 2:**
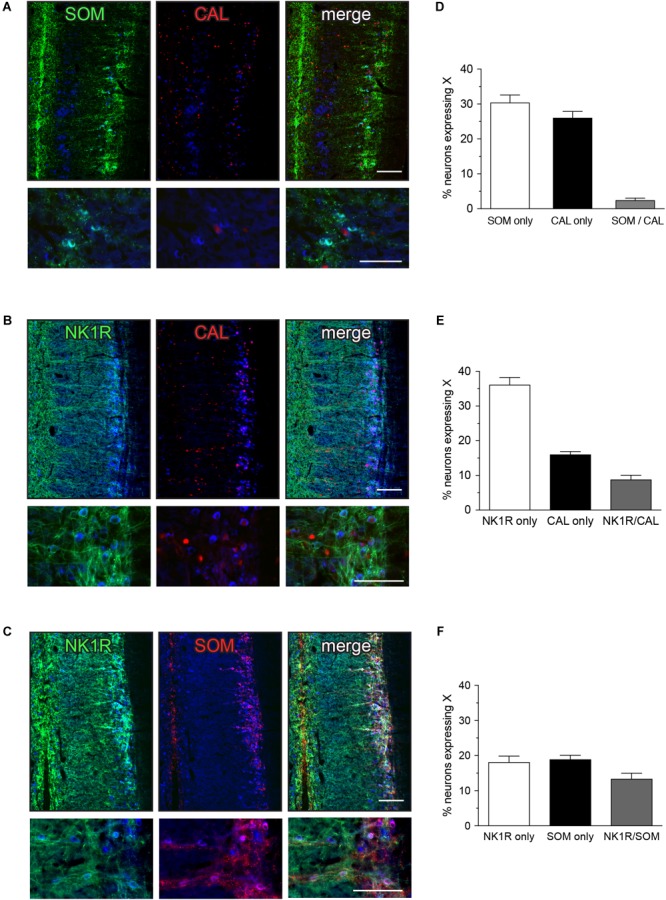
Expression of somatostatin (SOM), calbindin (CAL), and neurokinin-1 receptor (NK1R) in L6-S1 preganglionic neurons. Representative horizontal sections of spinal cord immunolabeled for pairs of neural markers are shown in **(A–C)**; higher magnification images from different sections are provided in the corresponding lower panels. Preganglionic neurons were identified by ChAT-immunoreactivity, here colorized blue. Images are oriented with rostral at the top and lateral on the right. SOM-, CAL-, and NK1R-immunoreactivity were each identified in subpopulations of preganglionic neurons. Immunoreactivity for SOM and CAL was cytoplasmic, NK1R-immunoreactivity was primarily in the plasma membrane and CAL-immunoreactive preganglionic neurons showed cytoplasmic or nuclear labeling, or both. SOM- and NK1R-immunoreactive dendrites extended toward the midline, and also labeled many neuronal somata near the midline. CAL-immunoreactive somata, presumed interneurons, were found between the intermediolateral column and the midline; these consistently showed cytoplasmic labeling. **(D–F)** Analysis of expression patterns in preganglionic neurons. **(D)** Subpopulations of neurons expressed either SOM or CAL, but very few expressed both SOM and CAL (SOM/CAL) (*n* = 6 rats). **(E)** Subpopulations of neurons expressed either NK1R or CAL, with few expressing both NK1R and CAL (NK1R/CAL). In total, 45.0 ± 2.4% of sacral preganglionic neurons expressed NK1R and 24.9 ± 1.5% expressed CAL (*n* = 5 rats). **(F)** Subpopulations of neurons expressed either NK1R or SOM, with some neurons expressing both NK1R and SOM (NK1R/SOM). In total, 33.1 ± 1.3% of preganglionic neurons expressed NK1R and 33.0 ± 1.3% expressed SOM (*n* = 6 rats). Calibration bars represent 100 μm.

SOM-immunoreactive neurons are shown in Figures 2A,C. Approximately one-third of IML neurons expressed SOM (Figure 2C), with the immunoreactivity localized in small clumps within the cytoplasm of a subgroup of neurons. This subcellular distribution resembled that commonly seen for neuropeptides in central and peripheral neurons. Many SOM-positive varicose axon profiles were present near the IML and SOM-positive neurons and varicose axon profiles were also clustered near the midline. CAL expression is shown in Figures 2A,B. CAL-immunoreactivity was primarily in neuronal nuclei, within a subgroup of approximately one-quarter of IML neurons and a minority of neurons located between the IML and the midline; some neurons instead showed cytoplasmic labeling. NK1R expression is shown in Figures 2B,C. NK1R-immunoreactivity was observed in approximately 40% of IML neurons, where it was especially concentrated on the plasma membrane of the soma and proximal processes. NK1R-positive neurons of similar appearance were scattered throughout the gray matter and more concentrated near the midline. Many NK1R axons were also observed.

Quantitative studies showed that SOM and CAL were expressed in largely separate neuronal populations that together make up more than half of the L6-S1 IML neurons (Figure 2D). Specifically, we found that 30.4 ± 2.2% of preganglionic neurons only expressed SOM, 26.0 ± 1.9% only expressed CAL and a few preganglionic neurons expressed both SOM and CAL (2.5 ± 0.6%) (*n* = 6). This indicates that SOM and CAL were largely separate populations that together constitute more than half of the IML neurons (Figure 2D). NK1R was expressed by subpopulations of both SOM and CAL neurons (Figures 2E,F). As a proportion of all preganglionic neurons, 8.8 ± 1.2% of preganglionic neurons expressed both NK1R and CAL (*n* = 5) and 13.4 ± 1.6% expressed both NK1R and SOM (*n* = 6).

### ATF3 Expression Is Upregulated Similarly in Each Molecular Subclass of IML Neuron

**FIGURE 3:**
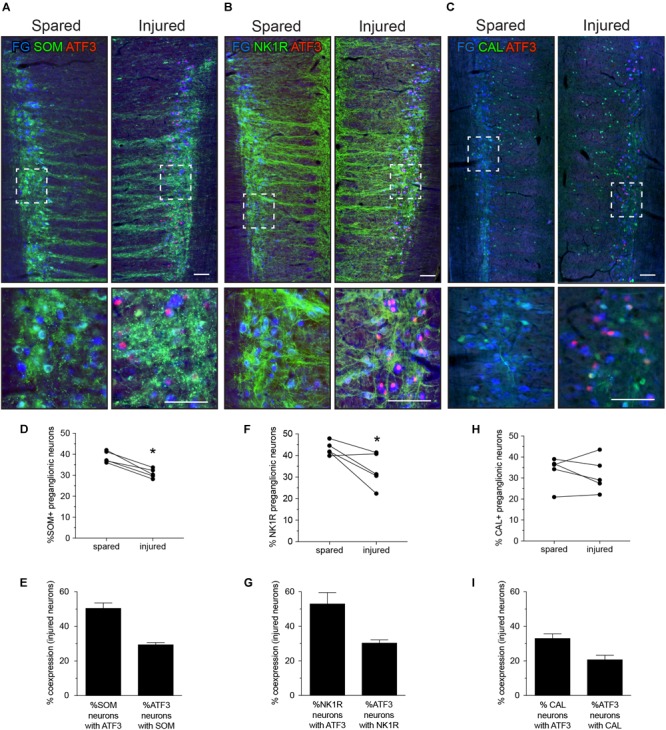
Expression of ATF3 in somatostatin (SOM), neurokinin-1 receptor (NK1R), and calbindin (CAL) subpopulations of L6-S1 preganglionic neurons one week after unilateral transection of the pelvic nerve. Representative horizontal sections of spinal cord immunolabeled for SOM, NK1R, or CAL (green) or ATF3 (red) are shown in **A–C**; higher magnification images from the same images are provided in the lower panels of each, as indicated by the boxes. Preganglionic neurons were identified by their uptake of retrograde tracer [FluoroGold (FG), here colorized blue]. Images are oriented with rostral at the top and lateral on the left (spared) or right (injured) of the relevant panel. Calibration bars represent 100 μm. ATF3 was only expressed in neurons on the injured side of the cord. **(D)** Injury reduced the proportion of preganglionic neurons expressing SOM (paired two-tailed t-test, *P* = 0.003; n = 5 rats). **(E)** Around half of the injured SOM-positive preganglionic neurons expressed ATF3; these comprised around one-third of the entire ATF3-positive population. **(F)** Injury reduced the proportion of preganglionic neurons expressing NK1R (paired two-tailed *t*-test, *P* = 0.043; *n* = 5 rats). **(G)** Around half of the injured NK1R-positive preganglionic neurons expressed ATF3; these comprised around one-third of the entire ATF3-positive population. **(H)** No effect of injury was detected on the proportion of preganglionic neurons expressing CAL (paired two-tailed *t*-test, *P* = 0.542; *n* = 5 rats). **(I)** Around half of the injured CAL-positive preganglionic neurons expressed ATF3; these comprised around one-third of the entire ATF3-positive population.

We then examined the impact of axotomy on L6-S1 IML populations visualized by FG and each of these markers (SOM, CAL, and NK1R) (Figure 3). No effect of the axotomy was evident by visual inspection of the pattern of immunolabeled sections, but the diversity of structures labeled by each antibody and the differences in axonal patterning between IML sections could easily obscure an effect on the preganglionic neuronal population.

Fewer preganglionic neurons were immunolabeled for SOM or NK1R after axotomy (Figures 3D,F), but no consistent effect on CAL neurons was detected. Because there is no strong evidence for death of these or thoracic preganglionic neurons within a week of axotomy (Peddie and Keast, 2011; Coulibaly et al., 2013), these new observations were interpreted as a reduced expression of SOM and NK1R, such that these neurons are no longer visible. Therefore, further analysis of this experimental data set is limited to those neurons that have retained detectable levels of SOM and NK1R after injury.

By analyzing expression of ATF3 in each class of L6-S1 IML neurons, we found that around half of the SOM and NK1R neurons expressed ATF3 after axotomy—i.e., a profile of ATF3 expression comparable to the total IML population (Figure 1C). From the earlier coexpression study (see above and Figure 2), it is plausible that these include neurons that express both SOM and NK1R. ATF3 was also expressed in about one-third of the CAL-positive IML neurons after axotomy. Conversely, about one-third of ATF3-immunoreactive neurons expressed SOM, another third expressed NK1R (which could potentially include SOM neurons) and around 20% expressed CAL. This suggested that around 20% of the ATF3-immunoreactive neurons do not express any of the other three markers. In summary, injury stimulated ATF3 expression in a diverse group of neurons.

### Hsp25 Is Upregulated by Axotomy

**FIGURE 4:**
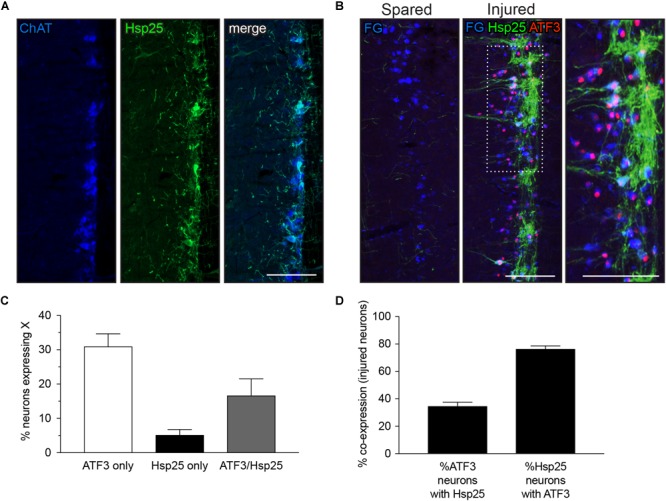
Expression of heat shock protein 25 (Hsp25) in lumbar and sacral preganglionic neurons. **(A,B)** Representative horizontal sections of spinal cord, oriented with rostral at the top and lateral on the right. **(A)** Preganglionic neurons in the intermediolateral column of the thoracolumbar spinal cord (T13-L2) were identified by immunoreactivity for choline acetyltransferase (ChAT, blue); a subpopulation of these neurons showed strong immunoreactivity for Hsp25 (green). **(B)** In the L6-S1 spinal cord, many preganglionic neurons (identified by FluoroGold labeling, colorized blue here) showed strong immunoreactivity for Hsp25 one week after axotomy; many of these Hsp25-immunoreactive neurons also expressed ATF3. In contrast, the uninjured (spared) side of the L6-S1 IML expressed neither Hsp25 nor ATF3. **(C,D)** Quantitation of L6-S1 preganglionic neurons after axotomy, showing that the majority of neurons upregulating Hsp25 also express ATF3, and that about one-third of ATF3 neurons showed upregulation of Hsp25 (*n* = 6). Calibration bars represent 100 μm.

Hsp25 has previously been reported in thoracolumbar preganglionic neurons of rodents (Plumier et al., 1997). We also identified strong Hsp25-immunoreactivity in thoracolumbar preganglionic neurons and axons in nearby regions (Figure 4A). Our initial visualization of the L6-S1 spinal cord of naive rats (data not shown) or the spared side of the L6-S1 cord, seven days after unilateral pelvic nerve injury (Figure 4B, left panel), failed to reveal any Hsp25 positive neurons, although some Hsp25-immunoreactive axonal processes were evident. In contrast, on the injured side, there was robust expression of Hsp25 in many IML neurons and neuronal processes (Figure 4B, middle and right panels). In contrast to the other neuronal markers discussed above, we found that the majority of Hsp25-positive IML neurons expressed ATF3 after axotomy (76.4 ± 2.3%, *n* = 6; Figures 4C,D).

## Discussion

In this study we have identified a heterogeneous response to axotomy by several neurochemically distinct populations of sacral parasympathetic preganglionic neurons in male rats, as summarized in Table 1. The primary outcome measure was upregulation of ATF3 expression measured within the first week after injury, which occurred in many but not all preganglionic neurons expressing SOM, NK1R, and CAL. Upregulation of ATF3 was more commonly observed in preganglionic neurons expressing Hsp25, which itself was upregulated by axotomy. Together these data reveal a new aspect of complexity within sacral preganglionic neurons and raise the possibility of diverse regenerative capacity and mechanisms.

**Table 1:** Impact of axotomy on major classes of sacral preganglionic neurons.

Neuronal class by marker(s)	% All preganglionic neurons	Expression of marker after axotomy	Expression of ATF3 in neuron class after axotomy
SOM ± NK1R	30.4 ± 2.2% SOM	Decrease in SOM	50.6 ± 3.0% SOM neurons
	33.0 ± 1.3% SOM	Decrease in NK1R^a^	53.1 ± 7.2% NK1R neurons^a^
CAL ± NK1R	26.0 ± 1.9% CAL	No change in CAL	33.1 ± 2.6% CAL neurons
	24.9 ± 1.5% CAL	Decrease in NK1R^a^	53.1 ± 7.2% NK1R neurons^a^
Hsp25	21.8 ± 2.5%^b^	Increase	76.4 ± 2.3%

*Neurons expressing both SOM and CAL were scarce so for summary purposes they are shown as separate classes of neurons. The proportion of preganglionic neurons expressing SOM or CAL was determined in two sets of NK1R coexpression experiments, so both results are shown here in column 2.*

*^*a*^It is not known if the axotomy-induced decrease in NK1R-immunoreactivity and upregulation of ATF3 in this neuron class occurs in SOM, CAL, or both NK1R-expressing classes, so the data have been repeated in each row. The potential coexpression of SOM, NK1R, and CAL by Hsp25 neurons was not investigated.*

*^*b*^Hsp25-immunoreactivity was only visible after axotomy.*

All sacral preganglionic neurons are cholinergic and express ChAT and use acetylcholine as the primary transmitter at peripheral synapses in the pelvic ganglia. However, SOM, CAL, and NK1R are examples of markers that identify subclasses of these neurons in the spinal cord or differentiate their projecting terminals in pelvic ganglia (Sasek et al., 1984; Keast and de Groat, 1989; Dhami and Mitchell, 1991; Keast, 1991, 1994; Papka and McNeill, 1993; Jen et al., 1996; Pidsudko, 2014; Zacharko-Siembida et al., 2017). The specific function of classes of sacral preganglionic neurons identified by SOM, CAL, and NK1R in the current study remains to be determined, but in rat pelvic ganglia, preganglionic terminals containing SOM are more commonly associated with evacuative (bladder and bowel) pathways than pathways projecting to the penile cavernosum (Keast and de Groat, 1989). SOM inhibits transmitter release in some autonomic ganglia (Ikeda et al., 1987; Katayama and Hirai, 1989) but it has not been determined if it has this function in pelvic ganglia. Studies in the rat sympathetic system indicate that CAL is expressed in functionally distinct preganglionic pathways that innervate the secretomotor neurons projecting to the submandibular salivary gland and pilomotor neurons projecting to skin, but not neurons innervating brown fat or vasculature of skin, muscle, or salivary glands (Grkovic and Anderson, 1997). The relationship between CAL expression and functional specificity in sacral pathways has again not been determined. As the preferred receptor for substance P, the expression pattern of NK1R may indicate which neurons are specifically influenced by pathways expressing this tachykinin, originating either from the brainstem or sacral dorsal root ganglia. We note that in thoracolumbar sympathetic preganglionic neurons, NK1R is more commonly expressed by neurons innervating the adrenal medulla or prevertebral ganglia, but is less commonly expressed by paravertebral ganglion neurons (Grkovic and Anderson, 1996). The function of NK1R sacral preganglionic neurons has not yet been determined.

The partial coexpression patterns of these three neural markers also point to the existence of a more complex combinatorial system for distinguishing functional classes. Their role in determining vulnerability to injury and capacity to regenerate also remains to be determined. CAL in particular has been implicated in neuronal responses to injury (Krebs et al., 1997; Dassesse et al., 1998; Obal et al., 2006). It is also noted that the mixed nature of our CAL labeling (exclusively nuclear in many neurons) may not fully reveal the extent of bioactive CAL distribution.

We have previously found that axotomy causes upregulation of ATF3 and c-Jun in only around half of the sacral preganglionic neuron population (Peddie and Keast, 2011), raising the question of whether these neurons, which may have distinct responses to injury and regenerative capacity, have additional specific neurochemical features. Using NK1R, SOM, and CAL, we were unable to identify a particular pattern of coexpression with ATF3, as a comparable proportion of each showed ATF3 upregulation after axotomy. We were also unable to detect any relationship between soma size and the upregulation of ATF3. If such a relationship were identified, it would by itself be difficult to interpret in a functional context, as visualizing ATF3 at a single point in time does not distinguish neurons that were originally smaller (at the time of injury) from those that became smaller after injury.

In the current study we focused primarily on ATF3 to indicate a classical “injury response,” again documenting that like the c-Jun upregulation, this occurred in around only half of the injured preganglionic neurons. Here, we further showed that upregulation of c-Jun occurred in many but all of the ATF3-immunoreactive neurons. The incomplete alignment of ATF3 and c-Jun expression following axotomy was not expected but may provide further indication of heterogeneous injury responses between different functional classes of preganglionic neurons or, if ATF3 and c-jun have distinct temporal expression and metabolic profiles, be an outcome of sampling tissue at a single time point. Investigating this at earlier time points after injury may be informative.

The apparent absence of ATF3 or c-Jun in so many neurons one week after axotomy is consistent with our earlier study on axotomized sacral preganglionic neurons (Peddie and Keast, 2011) and a study by Isaacson and colleagues on axotomized thoracic preganglionic neurons (Coulibaly et al., 2013). The reason for this heterogeneity has not yet been determined. It may be relevant to consider the site of injury, as Mason and colleagues found that expression of ATF3 by corticospinal neurons was more pronounced when axotomy was performed within 300–500 μm of the soma, with less ATF3 expressed after more distal lesions (Mason et al., 2003). The experimental injury performed in the current study was quite distant (several millimeters from the somata of sacral preganglionic neurons), raising the possibility that an injury closer to the spinal cord would initiate upregulation of ATF3 in a larger population of neurons. The more distal injury that we chose may have been a more sensitive way to reveal heterogeneity between different sacral preganglionic neurons.

In the current study we found that sacral preganglionic neurons differ from more rostral, sympathetic preganglionic neurons (Costigan et al., 1998) in their absence of detectable Hsp25 expression. This heat shock protein (human homolog Hsp27) has been implicated in injury responses in the sensory system (Costigan et al., 1998; Lewis et al., 1999). To our knowledge its function has not yet been investigated in autonomic preganglionic pathways. In addition to the unexpected absence of Hsp25 from uninjured sacral preganglionic neurons, its upregulation from an undetectable level to intense expression within ∼20% of sacral preganglionic neurons following axotomy was unexpected. Of particular interest, ∼80% of these Hsp25-immunoreactive injured neurons expressed ATF3—a much broader ATF3 response than the other neuronal classes or the overall population of sacral preganglionic neurons. Understanding the specific molecular and regenerative features of this neuronal population is of particular interest in the future, especially as there is evidence that ATF3 expression and JNK activation are necessary for Hsp25 induction (Nakagomi et al., 2003).

There are several technical limitations in our study. First, we only studied one time point after injury, although this was chosen based on earlier studies that showed one week as being optimal for identifying several changes in axotomized preganglionic neurons (Peddie and Keast, 2011; Coulibaly et al., 2013). Second, the reduced expression of NK1R and SOM by some preganglionic neurons, to the point that they could no longer be visualized, provides a relatively crude measure of a change in expression; many other neurons could have had reduced expression but were still visible by immunohistochemistry. The visual loss of neurons that previously expressed SOM and/or NK1R also impacted on the resolution at which we could interpret quantification of ATF3 expression in each population. We also note that cutting the pelvic nerve not only injures sacral preganglionic neurons but also injures sensory axons from sacral dorsal root ganglion neurons and some sympathetic axons from the sacral paravertebral chain ganglia that project through this nerve. Therefore, other areas of spinal cord and peripheral circuitry will also likely be undergoing change during this first week after injury.

In summary, our studies reveal the molecular complexity of sacral preganglionic neurons and their responses to injury. The simultaneous upregulation of Hsp25 and ATF3 may indicate a distinct mechanism of regenerative capacity after injury. A priority is now to define the function of these neuronal populations and to determine if their capacity for regeneration is indicated by their immediate early gene expression.

## Author Contributions

JK conceived and designed the study. AW conducted the experiments. AW, PO, and JK contributed to data analysis and interpretation, contributed to writing the manuscript, and all read and approved the submitted version.

## Conflict of Interest Statement

The authors declare that the research was conducted in the absence of any commercial or financial relationships that could be construed as a potential conflict of interest.
